# Periodontal Dressing: A Review Article

**DOI:** 10.5681/joddd.2013.040

**Published:** 2013-12-18

**Authors:** Zahra Baghani, Mahdi Kadkhodazadeh

**Affiliations:** ^1^Assistant Professor, Shahid Beheshti University of Medical Sciences (International Branch), Tehran, Iran; ^2^Associate Professor, Shahid Beheshti University of Medical Sciences, Tehran, Iran

**Keywords:** Biocompatibility, Coe-Pak, cytotoxicity, periodontal dressing, therapeutic agent, wounds

## Abstract

The purpose of this paper was to review the commercially available periodontal dressings, their physical and chemical properties, biocompatibility and therapeutic effects. Electronic search of scientific papers from 1956 to 2012 was carried out using PubMed, Scopus and Wiley InterScience search engines using the searched terms periodontal dressing, periodontal pack. Numerous *in vitro* and *in vivo* studies have evaluated various properties of periodontal dressings. Physical and chemical properties of dressings are directly related to their dimensional changes and adhesion properties. Their biocompatibility and therapeutic effect are among the other factors evaluated in the literature. Chlorhexidine is the most commonly used antibacterial agent in studies. In general, when comparing the advantages with the disadvantages, application of periodontal dressing seems to be beneficial. Numerous factors are involved in selection of an optimal dressing such as surgeon&rsquo;s intention, required time for the dressing to remain on the surgery site and its dimensional changes.

## Introduction

Periodontal dressings were first introduced by Dr. A.W Ward in 1923, who suggested the use of periodontal dressing following periodontal surgery. Periodontal dressings are now widely used for various purposes by periodontists, although some controversy exists regarding the necessity of their application following periodontal surgery.^1,2^

In some cases use of periodontal dressing is really beneficial. Protecting the wound from mechanical trauma and stability of the surgical site during the healing process are among the most important advantages of periodontal dressing application after surgery.^3,4^Other advantages include: patient comfort during tissue healing after surgery, good adaptation to underlying gingival and bone tissue, prevention of post-operative hemorrhage or infection, decreasing tooth hypersensitivity in the first hours after surgery, protecting the clot from the forces applied during speaking or chewing, preventing gingival detachment from the root surface,^5^ prevention of coronal flap displacement in apically repositioned flaps, providing additional support in free gingival grafts, and the last but not the least^6^protection of denuded bone during the healing process and splinting of mobile teeth after surgery. In non-surgical procedures, use of periodontal dressing can be helpful in aggressive periodontitis patients.^7^

However, despite all the aforementioned advantages, indications for use of periodontal dressings are limited. The present study is a literature review to assess the clinical application of periodontal dressings.

##  Method

This study was a structured literature review of articles published from 1956 to 2012.

PubMed (http://www.ncbi.nlm.nih.gov/pubmed/), Scopus (www.Scopus.com), and Wiley InterScience databases were used to search periodontal pack and periodontal dressing key words.

**Figure 1.**
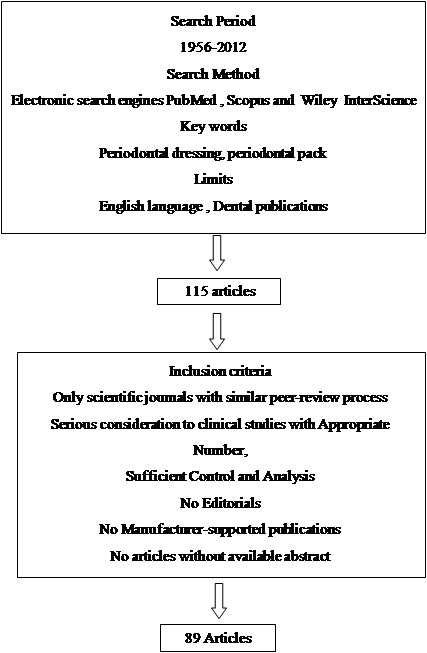
Literature search criteria.

The search was limited to English language publications. Searching of key words limited to dental publications yielded a total of 116 results. By application of inclusion criteria the obtained results further reduced to 83 citations that formed the basis for this review (Figure 1).

### Physical and Mechanical Properties

Only a limited number of studies have evaluated the physical and mechanical properties of periodontal dressings. These properties depend on the composition of periodontal dressing. To date, there is no exact and standardized reproducible technique to evaluate these properties. In addition, regarding new periodontal dressings, there is not sufficient research available.

Periodontal dressing material should be slow-setting to allow manipulation and to create a smooth surface causing no irritation, should be flexible enough to withstand distortion and displacement, should be adhesive and coherent without being bulky, and must have dimensional stability to prevent salivary leakage and plaque accumulation.^11,12^Evaluation of physical properties is valuable because these properties can affect the material’s clinical behavior, including its adaptation to the underlying tissues, which is directly related to dimensional changes and its adhesion properties to gingiva and tooth.^13^Assessment of dimensional changes is also beneficial because improved adaptation (less dimensional changes) decreases the accumulation of plaque under the dressing.

Gjerdet evaluated the dimensional changes of three currently available periodontal dressings after setting (Coe-Pak, Ward’s Wondrpak and Peripac).

All the dressings showed contraction during the first minutes after completion of their setting. This contraction culminated in Peripac at approximately 40 minutes and after about 2.5 hours the dressing exhibited expansion; however, the contraction continued at a slower pace in other products. Thus, greater dimensional changes that occur in Peripac can be harmful, leading to the distortion of surgical area.^13,14^

The dimensional changes of Coe-pak, Ward’s Wondrpak and Coe-Pak Hard and Fast Set periodontal dressings were evaluated in another study. As expected, contraction occurred in three materials after mixing but it was more significant in Ward’s Wondrpak than in other products and continued for 24 hours.^10^

Another physical property evaluated in the study mentioned above was working and setting times. Working and setting times differ based on the composition of the dressing, and have been assessed in a limited number of studies.^10^This study showed that Ward’s Wondrpak had a significantly longer working time than the other two products, but no significant differences were found between the two Coe-Pak products. Setting time of Ward’s Wondrpak under oral conditions was 24 minutes which was shown to be less than its working time under room conditions because both heat and moisture accelerate the reaction of ZOE.

Another physical property evaluated in studies was the adhesive rate of periodontal dressing to both gingiva and tooth. This property is especially important considering its role in prevention of microbial penetration. Studies have used two methods to increase the retention of periodontal dressings.^15,16^

The first method is by application of dressing into interdental spaces to physically increase retention. By doing so, a rigid material is formed around the teeth after the completion of setting. Different means have also been described in studies to enhance retention such as wire, dental floss, acrylic compound, copper band, tin foil, etc.^16-18^ However, these tools have been shown to result in weaknesses, leading to the failure of the dressing instead of fortifying it. The retention by splint and stent is like the other devices used. Ideally, the dressing should be sufficiently retentive without the need for additional devices.^19,20^

Different investigators have evaluated the adhesion properties of periodontal dressing by tensile and shear strength assessment.

Goldman and Cohen (1973) emphasized the need for a rigid and secure periodontal dressing with good adhesive properties.^12^They pointed out that this property would be achieved by adding polyacrylic acid and cyanoacrylate to the dressing material composition.^21^

Some studies have introduced new periodontal dressings like quaternary aluminum borate cement, suggesting that this material is worthy of investigation as a potential dressing material after the assessment of its tensile and shear strengths.^22^Several researchers have used cyanoacrylate without suturing and have protected the wound from bacterial invasion.^23^

In a comparative study, adhesive strength of various dressings to tissue (Coe-Pak Hard and Fast Set, Coe-Pak, and Ward’s Wondrpak) was evaluated. In Ward’s Wondrpak, shear and tensile adhesive bond strength to enamel was significantly less than the other product. In another study, adhesive properties of different dressings to enamel were evaluated (Coe-Pak, Peripac and Peripac Improved) and the three materials were found to have poor adhesive properties; however, Coe-Pak showed higher adhesive properties.^9^

### Clinical Studies

Surgical area is covered with periodontal dressing for 3-14 days following periodontal surgery whenever necessary. It has been reported that the dressing accelerates the healing process, but a general consensus has not been reached on the necessity of application of periodontal dressing on periodontal wounds. In a study, it was concluded that dressing per se can cause little damage to the normal periodontium, but in the long term, inflammation increases because of plaque accumulation under the dressing.^24^

Assessment of plaque indexes after the application of dressing, apart from the surgical technique, revealed no significant differences between the test and the control groups.^18,25,26^

Less plaque accumulation was observed when a light-cured periodontal dressing (Barricaid) was used, but no differences were found in clinical indexes.^27^ Barricaid has been used in specific surgical and orthodontic procedures as well.^28,29^

Another study evaluated clinical indexes after reversed bevel flap and found no significant differences in gingival fluid assessment among groups. However, the situation was reversed for gingival index assessment. On day 7, the undressed area showed more bleeding and sensivity.^1^

It seems regarding the differences in the method of clinical studies evaluation of the definite effect of periodontal dressings on the clinical indexes is not possible. Overall, there were no statistically significant differences in clinical indexes.

The effect of periodontal dressing on pain and the amount of analgesics taken by the patient is another factor that has been evaluated in the literature. In a study on the amount of analgesics taken by the patients, apart from the surgical technique, no statistically significant differences were detected in this respect between the dressing and non-dressing groups;^30^ however, no other study has confirmed this result.^31^Haugan et al compared Peripac with another commercially available dressing and reported that patients in the Peripac group showed significantly more pain, swelling, and inflammation,^32^but no significant differences were detected in this respect in some other studies.^30^

In another study on pain severity after gingivectomy, two periodontal dressings (Coe-Pak, Ward’s Wondrpak) and different local anesthetic agents were compared. They showed that the local anesthetic combination of lidocaine-adrenalin (1:80,000) results in a higher mean post-operative pain experience after gingivectomy. Eugenol present in Wondrpak is responsible for less pain experience reported in this group due to its analgesic properties.

Haugan et al (1973) evaluated three periodontal dressings (Coe-Pak, Peripac, Ward’s Wondrpak) in terms of pain, swelling, bleeding and use of analgesics in patients. These criteria were higher in Peripac group, but no statistically significant differences were reported in another clinical study. Greater pain experience in the test group (with dressing) seems to be due to plaque accumulation under periodontal dressing and subsequent microbial invasion. As a result, reduction of microbial contamination can lead to wound healing and less pain.^33^

In addition, better wound healing has been reported after using chlorhexidine in periodontal dressings.^34^However, Peripac had no antibacterial effect on salivary bacteria after setting.^35^Clinically visible plaque accumulations under the dressing has been reported by numerous researchers.^36,37^The present inflammatory reaction can be explained by the presence of microorganisms, and physical and chemical properties of dressing are important as well.^35^

### Biocompatibility of Periodontal Dressings

**Figure 2.**
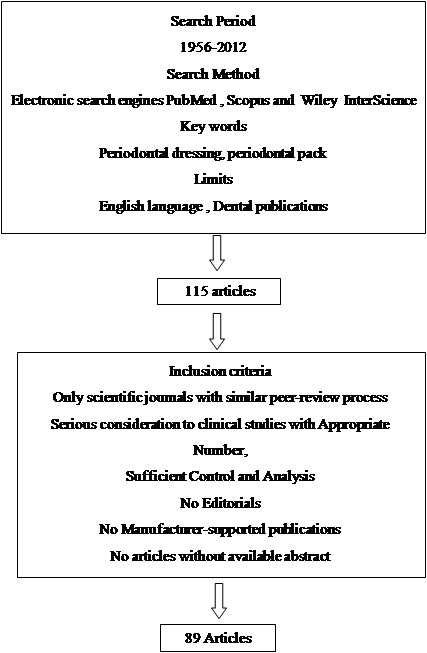
Studies assessing periodontal dressing cytotoxicity.

A wide range of materials are used in dentistry that can cause allergic reactions in the oral cavity, although due to the presence of saliva and the vascularization of the oral mucosa, prevalence of allergic reactions in the oral cavity is less than that on the skin. Contact stomatitis as the result of application of periodontal dressing has been frequently reported in the literature.^38-41^ Some in vitro tests have been introduced to evaluate the cytotoxicity of dressings by cell media.^42^ Implantation tests have also been used to assess local cytotoxicity.^43-45^Many cells from human and animals have been used to monitor the cytotoxicity of dressings(Figure 2).^46-48^

### Therapeutic Effects of Periodontal Dressings

Previously, periodontal dressings used to be applied to cause gingival shrinkage in cases where surgery was medically or psychologically inadvisable. Also, Orban (1943) described a technique of chemosurgery by using paraformaldehyde in a dressing.^60^

Thus, the therapeutic effects of substances used in the composition of dressings after periodontal surgery has been the aim of numerous investigations. These materials are classified into two categories:

1. Agents with effects on oral bacteria
2. Agents with effects on periodontal tissues

In this regard, several agents have been added to the composition of periodontal dressings such as: tetracycline, zinc bacitracin, non-eugenol phenol derivatives, chlorothymol, oil of bergamot and chlohexidine.^61-65^

Steroids and Dilantin were also added to facilitate and accelerate tissue healing. It is important to note that chemical inactivation of the materials added may occur during the process.^66,67^

In some limited research studies, surgical side effects, like root hypersensitivity, were also improved by adding some agents. Besides, new materials such as cyanoacrylate have been introduced as a substitute for periodontal dressing.^68^

### Evaluation of Microorganisms and Antibacterial Properties

**Figure 3.**
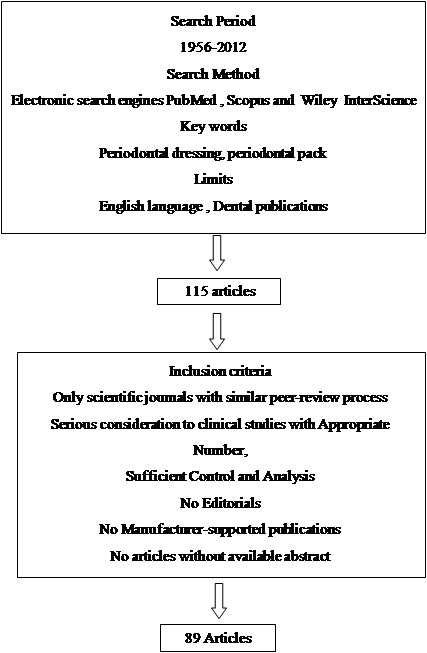
Microorganisms evaluation and antibacterial properties of periodontal dressings

The results of these studies are summarized in Figure 3.

### Other Properties

Addition of other agents to periodontal dressing with different properties was also evaluated by some researchers. Of these materials we may name two synthetic pyrimidine compounds (MS-430, MS-818) that have been added to Coe-Pak. These ingredients can accelerate angiogenesis. The effect of MS-818 on the formation of tubule-like structures was higher. The solubility of MS-818 was less than MS-430 in the medium and the MS-818 reinforced the effect of VEGF on the endothelial cells.^82^

### Review of Different Periodontal Dressings

Periodontal dressing composition has changed during the years; at present, these materials are divided into the following three categories:

1. Those containing zinc oxide and eugenol
2. Those containing zinc oxide without eugenol
3. Those containing neither zinc oxide nor eugenol

### Periodontal Dressings Containing Zinc Oxide and Eugenol

#### Ward's Wondrpak

This product was marketed in the form of powder and liquid (the product is no longer produced commercially). The liquid contains eugenol, rose oil or peanut and resin. The powder contains zinc oxide, powdered resin and tannic acid. The powder and liquid are mixed on paper pad, and then the prepared paste is used immediately or is wrapped in aluminum foil to be frozen for one week.^6^

### Periodontal Dressings Containing Zinc Oxide without Eugenol

#### Coe-Pak

The reaction between a metallic oxide and fatty acids is the basis for Coe-Pak (*De Trey/Denstply, Konstanz, Germany*). It is supplied in two tubes, the contents of which are mixed immediately before use. One tube contains zinc oxide, oil, a gum, and lorothidol. The other tube contains liquid coconut fatty acids thickened with colophony resin and chlorothymol.^8^

#### PeriPac

PeriPac (GC America Inc., Chicago, USA) is supplied as one paste, and is composed of calcium sulfate, zinc sulfate, zinc oxide, polymethylmethacrylate, dimethoxytetra-ethylene glycol, ascorbic acid, flavor and iron-oxide pigment. To use this material, a small quantity should be taken from the jar with a dry sterile spatula and deposited on a paper napkin.

Medications in powder form can be added if desired. Hardening of Peripac begins as soon as it comes into contact with water and is complete in about 20 minutes. Application of the dressing should not take more than 2-3 minutes. A correctly applied dressing remains with no change for 8-10 days.

One advantage of this material is to treat necrotic gingivitis. In such cases an antibiotic powder should be added by rolling it into the material on the paper napkin. The dressing keeps the medicament in contact with the ulcerated area. Protection of non-specific lesions or sutured margins, fixation of dressing medicaments to cervical area and temporary rebasing of immediate dentures in periodontal surgery are among other indications of this paste.^83^

#### Vocopac

Vocopac (Voco, Cuxhaven, Germany) is supplied as two pastes (base and catalyst) that cure chemically. This material remains elastic in the patient^’^s mouth and is not brittle. Vocopac contains purified colophonium, zinc oxide, zinc acetate, magnesium oxide, fatty acids, natural resin and natural oils and colorant e127. Its use is contraindicated in patients who are allergic to these ingredients and contact with the bone should be avoided as well. Slight discoloration of synthetic materials may also occur.^84^

#### SeptoPack

This product (*Septodont, saint-maur-des-fosses cedex, France*) is supplied in 60-g jars. The composition of this product includes amyl acetate, dibutyl phthalate (10-25%), methyl polymethacrylate, zinc oxide (20-50%) and zinc sulfate (2.5-10%). This product is a self-setting plastic paste containing fibers in its mass. Working time in the mouth is only 2 or 3 minutes following application. Setting time is about 30 minutes.

This product contains dibutyl phthalate which is very toxic to aquatic organisms. This product may harm the eyes in an unborn child and has possible risk of impaired fertility. Therefore, protective clothing, gloves and respiratory equipment are mandatory.^85^

#### Periocarea 

This product (*Voco, Cuxhaven, Germany*) is supplied in two tubes (paste and gel). Equal amounts of paste and gel must be mixed on the mixing pad until the color becomes uniform. Setting time of this product is 45-60 seconds and the working time is 4-5 minutes.^86^

### Periodontal Dressings Containing neither Zinc Oxide nor Eugenol

This group includes cellulose-based periodontal dressings like Reso-pac and Mucotect.

#### Reso-pac

This product (*Hager & Werken Gm bH & Co. KG, Post fach, Germany*) is supplied as one hydrophilic paste and is ready for use without mixing. This dressing remains in place for up to 30 hours, even on bleeding wounds, because of its hydrophilic properties. Reso-pac swells up to a gel-like consistency after about 3 minutes.^87^

#### Mucotect

This product (*Hager & Werken Gm bH & Co. KG, Germany*) is supplied in one tube and contains carboxymethyl cellulose, polyvinyl acetate, ethyl alcohol, vaseline and polyethylene oxide resin. Mucotect is a hydrophilic paste and adheres to the area for up to 30 hours. Due to its composition, it adheres very well to damp and even bleeding areas.^88^

#### Barricaid 

Barricaid (*Pupdent, watertown, USA*) is available in a syringe for direct placement. The syringe is also suitable for an alternate indirect technique. A visible light-curing unit is required for the setting of this dressing.

This product has a translucent character which provides superior esthetics. Barricaid is mainly composed of polyether dimethacrylate, silanized silica, accelerator, VLC photo-initiator and colorant.^89^

### Materials Compared with Periodontal Dressings in the Literature

Some studies have used different materials as dressing such as carboxymethyl cellulose, aluminum borate, Myzotect-Tincture and fittydent (denture adhesive cream to increase retention). Adhesion to soft tissue is the reason for their application for this purpose, and there is no brand name to label them as periodontal dressing. Cross-Pack is another material which has just been used in one article, and English literature search on this product yielded no results.

##  Conclusion

Physical properties, availability, biocompatibility and therapeutic effects of periodontal dressings were briefly discussed in this review article.

Use of periodontal dressings after surgery seems beneficial. But, it would be better to limit their application to specific cases; for example, their application is not necessary in undisplaced flaps where the flap returns to its previous position and gingival bleeding and root hypersensitivity are minimal.

Overall, when the advantages outweigh the disadvantages, application of periodontal dressing would be beneficial. Multiple factors are involved in selection of the dressing of choice, such as:

Surgeon’s aim of using periodontal dressing
Required time for periodontal dressing to remain on the surgical area: long-term application of Coe-Pak may increase its cytotoxic effects. Ward’s Wondrpak is more cytotoxic than other products and Barricaid is cytocompatible when its polymerization is complete. It seems that cellulose-based periodontal dressings lead to less inflammatory reactions and are probably more acceptable by the patient.
Dimensional changes: All dressings have weak adhesive properties. Thus, plaque accumulates under them and decelerates the healing process. Based on the literature, Peripac and Ward’s Wondrpak have the greatest dimensional changes, although the other dressings have not been thoroughly evaluated in this respect.

Choosing an optimal periodontal dressing is a difficult decision to make because they have to be compared under equal conditions.

In general, it seems that cellulose-based periodontal dressings can replace the traditional dressings. In terms of therapeutic effects, the expected success is not always achievable by changing the physical properties of therapeutic agents.
